# Genetically engineered magnetic nanocages for cancer magneto-catalytic theranostics

**DOI:** 10.1038/s41467-020-19061-9

**Published:** 2020-10-27

**Authors:** Yang Zhang, Xiaoyong Wang, Chengchao Chu, Zijian Zhou, Biaoqi Chen, Xin Pang, Gan Lin, Huirong Lin, Yuxin Guo, En Ren, Peng Lv, Yesi Shi, Qingbing Zheng, Xiaohui Yan, Xiaoyuan Chen, Gang Liu

**Affiliations:** 1 State Key Laboratory of Molecular Vaccinology and Molecular Diagnostics, Center for Molecular Imaging and Translational Medicine, School of Public Health, Xiamen University, 361102 Xiamen, China; 2 State Key Laboratory of Cellular Stress Biology, Innovation Center for Cell Biology, School of Life Sciences, Xiamen University, 361102 Xiamen, China; 3 Yong Loo Lin School of Medicine and Faculty of Engineering, National University of Singapore, 117597 Singapore, Singapore; 4 Fujian Provincial Key Laboratory of Biochemical Technology, Institute of Biomaterials and Tissue Engineering, Huaqiao University, 361021 Xiamen, China; 5 Fujian Provincial Key Laboratory of Innovative Drug Target Research, School of Pharmaceutical Sciences, Xiamen University, 361102 Xiamen, China

**Keywords:** Biomimetics, Nanobiotechnology, Biomedical engineering, Biomaterials, Nanoparticles

## Abstract

The clinical applications of magnetic hyperthermia therapy (MHT) have been largely hindered by the poor magnetic-to-thermal conversion efficiency of MHT agents. Herein, we develop a facile and efficient strategy for engineering encapsulin-produced magnetic iron oxide nanocomposites (eMIONs) via a green biomineralization procedure. We demonstrate that eMIONs have excellent magnetic saturation and remnant magnetization properties, featuring superior magnetic-to-thermal conversion efficiency with an ultrahigh specific absorption rate of 2390 W/g to overcome the critical issues of MHT. We also show that eMIONs act as a nanozyme and have enhanced catalase-like activity in the presence of an alternative magnetic field, leading to tumor angiogenesis inhibition with a corresponding sharp decrease in the expression of HIF-1α. The inherent excellent magnetic-heat capability, coupled with catalysis-triggered tumor suppression, allows eMIONs to provide an MRI-guided magneto-catalytic combination therapy, which may open up a new avenue for bench-to-bed translational research of MHT.

## Abstract (web summary)

The clinical application of magnetic hyperthermia therapy (MHT) is limited by the poor magnetic-to-thermal conversion efficiency of MHT agents. Here, the authors develop encapsulin-produced magnetic iron oxide nanocomposites (eMIONs) with excellent magnetic-heat capability and catalysis-triggered tumor suppression ability to overcome the critical issues of MHT.

## Introduction

Magnetic hyperthermia therapy (MHT) is a new approach for minimally invasive treatment of cancer^1,2^ and has been tested in the clinic^3–5^. Among the various types of magnetic thermal induction agents, iron oxide nanoparticles (IONs) show some promise due to their safety and magnetic property (e.g., clinical contrast agents for magnetic resonance imaging (MRI))^6–8^. However, conventional superparamagnetic IONs suffer from low magnetic-to-thermal conversion efficiency^9–12^, and the highest reported specific absorption rate (SAR) of superparamagnetic IONs is only 487 W/g^4^, which is insufficient for effective MHT in the range of safe dosage in clinical application. Another issue is the poor stability of ferrimagnetic materials in the presence of an alternative magnetic field (AMF), which are less favorable for biomedical applications in spite of their relatively high heat-induction efficiency^3^. Therefore, it is imperative to develop IONs with optimized magnetic-to-thermal conversion efficiency and great biocompatibility for the clinical translation of MHT.

Recently, bionics technology such as biomineralization has drawn much attention to produce uniform, stable, and well-ordered IONs via precise control of ion concentration, nucleation process, pH, or redox potential in magnetic protein nanocages^13^. Various magnetic protein nanocages with ferroxidase centers (FOCs), functional proteins to facilitate mineralization, exist in bacteria, ranging from small DNA-binding protein from starved cells (Dps) cage (8 nm), mid-sized ferritin (12 nm), to the large virus capsid-like encapsulins (24 nm or 32 nm)^14–19^, but no efforts have been reported to investigate whether magnetic protein nanocages can be used as MHT agents. Interestingly, these protein nanocages offer a highly tunable environment and facile control of the composition, morphology, magneto-crystalline anisotropy, and magnetization of IONs. Therefore, we hypothesize that IONs derived from magneto-protein nanocages with improved heating efficiency may be able to overcome the current obstacles of MHT.

**Fig. 1:**
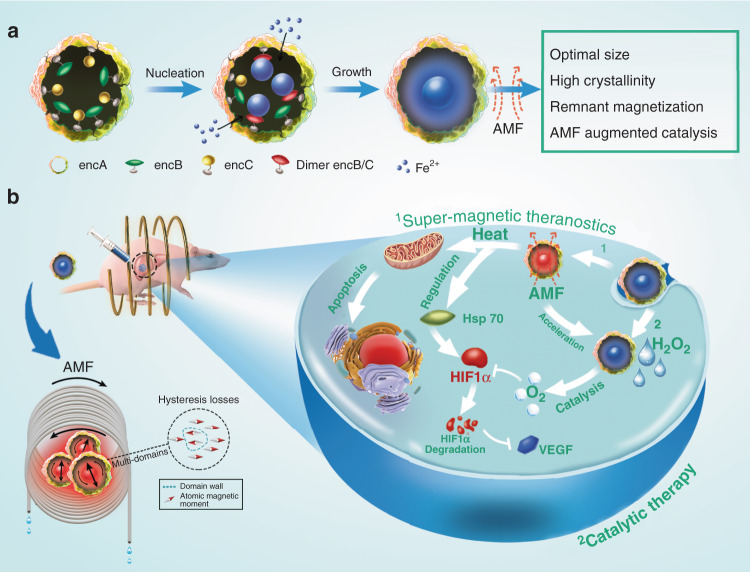
Schematic illustration of magneto-catalytic theranostics. **a** Schematic of an encABC and its biomimetic mineralized procedure. The inner cores of encapsulin-produced magnetic iron oxide nanocomposites (eMIONs) have the optimal size for magnetic hyperthermia induction and consist of FeO subdomain-containing Fe_3_O_4_, with ~100% crystallinity, and have remnant magnetization, thus achieving prominent magnetic-heat induction in an alternative magnetic field (AMF). Moreover, eMIONs are great catalase-like nanozymes that can be utilized for catalytic therapy. **b** eMIONs accumulated in tumor sites and suppressed tumor growth effectively, as monitored by multimodality imaging. eMIONs retained in the tumor region augmented oxygen production to modulate the tumor microenvironment, decrease HIF-1α, and VEGF expression. Then, at an optimal time point, AMF not only induced MHT but also improved the catalytic efficacy, resulting in a magneto-catalytic therapy (MCT).

Encapsulin is an iron-storage protein nanocage in *Myxococous xanthus*, featuring excellent biocompatibility, the appropriate size of the cavity, and FOCs^16^. In this study, we genetically engineered encapsulin protein nanocages to produce high-quality IONs via a biomineralization procedure (Fig. 1a). The encapsulin-produced magnetic IONs (eMIONs) consist of FeO subdomains containing Fe_3_O_4_ with ~100% crystallinity. Notably, eMIONs have excellent magnetic saturation and remnant magnetization to produce coercive force under AMF, which significantly improves the energy-dissipation rate to promote the magnetic-to-thermal conversion capacity^20,21^, achieving an ultrahigh SAR of 2390 W/g in AMF at 300 kHz and 15 kA/m. Moreover, eMIONs are excellent intrinsic catalase-like nanozymes and show enhanced catalytic activity in the presence of AMF to achieve synergy effects for MHT. The MRI-guided magneto-catalytic combination therapy (MCT) shows great tumor suppression and nearly doubled survival time of orthotopic hepatocellular carcinoma (HCC)-bearing mice. Therefore, eMIONs have great potential to be a clinically translatable MHT agent to overcome the crucial issues facing MHT (Fig. 1b).

## Results

### Engineering and characterization of encapsulin protein nanocage

**Fig. 2:**
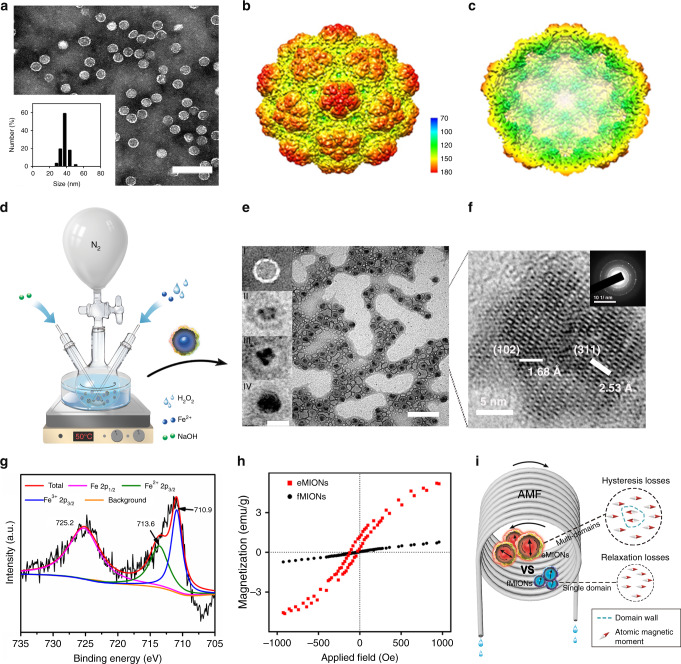
Characterization of encapsulin-produced magnetic iron oxide nanocomposites (eMIONs). **a** Representative TEM image of encABC (Inset: DLS of encABC). Scale bar: 100 nm. **b**, **c** Isosurface representation of reconstructed nanoparticles color-coded according to the radial distance from the center (given in Å): **b** view from the outside; **c** cutaway view of the internal structure. **d** Schematic diagram of the biomimetic synthesis of eMIONs and the heat emission under alternative magnetic field (AMF). **e** Representative TEM (scale bar: 100 nm) image of eMIONs (inset images I to IV: biomimetic synthesis time at 0, 1, 3, and 6 h, respectively). Scale bar: 50 nm. **f** HRTEM and corresponding FFT pattern (inset) of eMIONs nanocrystals, corresponding to {102}-faceted FeO and {311}-faceted Fe_3_O_4_. **g** XPS analyses of eMIONs. **h** The results of VSM analysis of eMIONs and fMIONs. **i** Schematic illustration of eMIONs and fMIONs for magnetic-to-thermal conversion under AMF. One of three repetitions with similar results is shown here for **a**, **e**, and **f**.

The recombinant encapsulin protein nanocage (encABC) was genetically engineered as follows: the shell protein encA was cloned into an expression cassette of pRSFDuet-1 plasmid, the inner functional proteins encB and encC were cloned into different expression cassettes of pCDFDuet-1 simultaneously (Supplementary Fig. 1a), and then the plasmids were co-transformed into *E. coli* BL 21 to obtain encABC. Cryo-electron microscopy (cryo-EM, Supplementary Fig. 1b) and transmission electron microscopy (TEM) confirmed that the purified material was composed of a homogeneous spherical particle about 32 nm in diameter (Fig. 2a). The recovered structure of encABC (Fig. 2b, c) showed the same structure as the native nanocompartment, featuring a hollow icosahedral symmetry structure made of 180 encA subunits with holes ~3 Å in diameter on its surface. Moreover, SDS-PAGE (Supplementary Fig. 1c) demonstrated that the particles are composed of three proteins: encA (35 kDa), encB (17 kDa), and encC (13 kDa). Gel quantitation estimated that the shell protein encA accounts for ~55% of the total protein content, and the inner function proteins encB and encC account for ~24% and ~21%, respectively. The expression quantity of functional proteins encB and encC in the nanocages is about two-fold higher than that of the native encapsulin^16^; thus we believed that the genetically engineered encABC would have much better biomineralization ability.

### Biomineralization and characterization of eMIONs for MCT

The size and crystalline degree of iron cores are essential for ION-based MHT^12^. Thus, the mid-sized ferritin magnetic nanocages were chosen as the control in our experiments. The encABC and ferritin nanocages were biomineralized to prepare the eMIONs and ferritin-produced magnetic IONs (fMIONs), respectively (Fig. 2d). TEM confirmed that purified eMIONs exhibited an electron-dense core (Fig. 2e and Supplementary Fig. 2a). Additionally, we found that the diameter of the iron cores of eMIONs (22 nm) was almost five times larger than that of fMIONs (4.5 nm), as measured by Image J (Supplementary Fig. 2c). TEM images also revealed that irons could be biomineralized to induce nucleation in the encABC nanocages, and then grew to form an iron core via a green biosynthetic procedure (Fig. 2e inset I–IV). It is also of note that eMIONs have remarkable mineralization ability, which enables much higher loading of Fe atoms (31,590 atoms/cage) compared with fMIONs (3394 atoms/cage) by weighing the protein and Fe of the nanocages. Dynamic light-scattering (DLS) analyses (Supplementary Fig. 2b) showed that the hydrate particle sizes of eMIONs and fMIONs were 39.3 ± 2.7 nm and 12.6 ± 1.2 nm, respectively. The stability of eMIONs is a key issue for further biomedical application. Thus, eMIONs were incubated into various temperature conditions (25, 37, 50, 60, and 70 °C), cell culture with 10% fetal bovine serum (37 °C) and cathepsin B (0.2 units, 37 °C) solutions for 48 h to investigate the thermal stability, blood stability, and protease degradation, respectively. As shown in Supplementary Fig. 2d–f, eMIONs were monodispersed without obvious aggregation/degradation, exhibiting excellent stability in all the conditions.

**Fig. 3:**
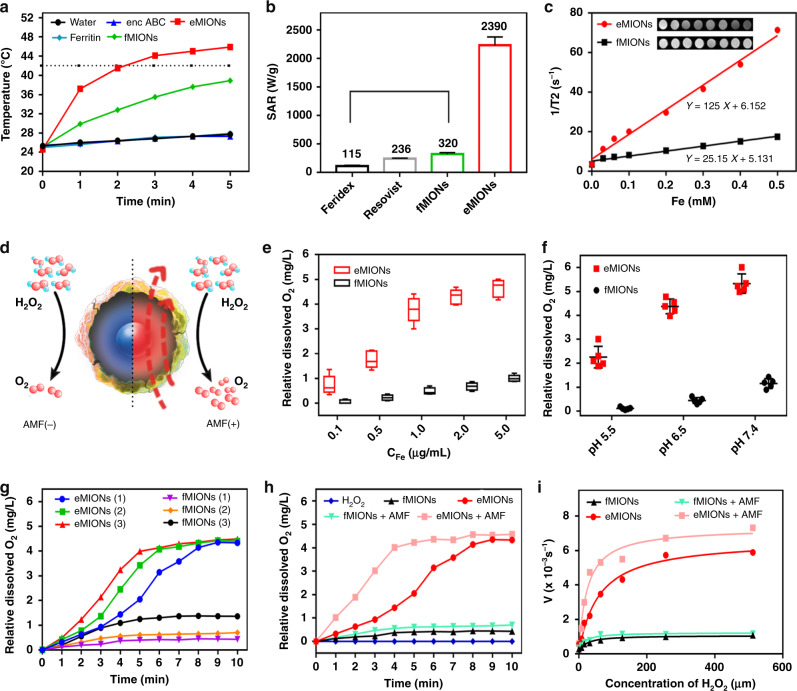
Functional properties of encapsulin-produced magnetic iron oxide nanocomposites (eMIONs). **a** Alternative magnetic field (AMF) induced temperature rise. Data are presented as mean ± SD (*n* = 5 independent experiments). **b** Comparison of SAR values of eMIONs to fMIONs and two types of commercial agents. **c** *T*_2_-weighted MR images and *T*_2_ relaxation rates (1/*T*_2_, s^−1^) of eMIONs and fMIONs at different iron concentrations. **d** Schematic illustration of eMIONs for enhancing catalysis under AMF. **e**–**g** The relative dissolved O_2_ in H_2_O_2_ (100 μM) after incubation of various concentrations of eMIONs and fMIONs (**e**, pH 6.8), various pH solutions (**f**), and various temperatures (**g**, 1 = 25 °C, 2 = 37 °C, 3 = 43 °C). Data are presented as mean ± SD (*n* = 5 independent experiments). Tukey boxplots are shown with the box indicating quartiles and the whiskers drawn at the lowest and highest points within 1.5 interquartile range of the lower and upper quartiles, respectively. Error bars show the 95% confidence interval for the difference in proportions or medians between the O_2_ production. **h**, **i** H_2_O_2_ (100 μM) decomposition by catalase-like activity of eMIONs with oxygen generation measured by the level of dissolved oxygen (**h**) and Michaelis–Menten kinetics (**i**) of eMIONs/fMIONs with/without AMF.

High-resolution TEM, XPS, and XRD analyses of IONs (Fig. 2f, g and Supplementary Fig. 3a–d) illustrated that the iron cores of eMIONs were FeO subdomains containing Fe_3_O_4_ (FeO@Fe_3_O_4_), while those of the fMIONs were mainly γ-Fe_2_O_3_. XRD data analyzed through JADE software demonstrated that the degrees of crystallinity of eMIONs and fMIONs were 99.99% and 47.66% (Supplementary Fig. 4), respectively. Therefore, we tested the magnetic field by a vibrating-sample magnetometer (VSM) (Fig. 2h and Supplementary Fig. 5). The result showed that the magnetization of eMIONs reached 15.3 emu/g (10 kOe, 300 K), which is nearly 6.5 times higher than that of commercial IONs Resovist (2.4 emu/g)^22^ and 7.5 times higher than that of fMIONs (2.0 emu/g). Notably, the results also revealed that eMIONs are multidomain nanoparticles, which have magnetic domains and remnant magnetization, suggesting that eMIONs could produce coercive force via hysteresis losses under AMF and significantly improve the energy-dissipation rate through a potent magnetic to heat induction^19,23,24^ (Fig. 2i). These differences between eMIONs and fMIONs might cause a huge gap in their intrinsic magnetic and catalytic abilities. Therefore, the heating capacities of MIONs were measured in AMF with different powers of 10–20 kA/m at 300 kHz and iron concentrations of 0.1–0.5 mM (Fig. 3a and Supplementary Fig. 6), from which the SAR values were calculated as well. As expected, eMIONs have an obviously higher SAR value (2390 W/g) as compared to fMIONs (320 W/g) and commercial IONs^5^, such as Feridex (115 W/g) and Resovist (236 W/g) (Fig. 3b). Also, the temperature of eMIONs increased rapidly to reach 42 °C in 5 min with AMF (15 kA/m, 300 kHz), but for fMIONs, it did not extend beyond 39 °C under the same condition, which is not surprising since eMIONs have a larger size, higher degree of crystallinity of iron cores and remnant magnetization to generate more heat in AMF than fMIONs^20^. It is also worth noting that the *r*_2_ value of eMIONs was 125 mM^−1^ s^−1^, whereas the *r*_2_ value of fMIONs was 25.15 mM^−1^ s^−1^ at 9.4 T (Fig. 3c). In general, higher *r*_2_ values of the IONs correspond with higher suitability of the contrast agent for *T*_2_-weighted imaging, suggesting that eMIONs have excellent sensitivity for MRI-guided MHT.

The catalase-like ability of eMIONs was investigated to evaluate its potential for catalytic therapy (Fig. 3d). Initially, the O_2_ production abilities of eMIONs and fMIONs were tested at different concentrations of Fe as well as different pH and temperature conditions (Fig. 3e–g), which indicated that the catalysis had an obvious positive correlation with all three parameters. As shown in Fig. 3h, the O_2_ production of eMIONs was 4.45 mg/mL, which was approximately eight times higher than that of fMIONs (C_Fe_ = 5 μg/mL, C_H2O2_ = 100 μM, pH = 6.8, 25 °C). More interestingly, the catalytic rate of eMIONs in AMF was ~44% higher. To better understand how the AMF affected the O_2_ production, we studied Michaelis–Menten steady-state kinetics (Fig. 3i). Accordingly, eMIONs had similar initial velocity (*V*_0_) with/without AMF as confined in varying H_2_O_2_ concentrations, but the maximum velocity (*V*_max_) was augmented by ~1.26 times under a magnetic field (15 kA/m, 300 kHz, C_Fe_ = 5 μg/mL, C_H2O2_ = 100 μM). Notably, the Michaelis constant (Km) of eMIONs in AMF was decreased by half, which means eMIONs exhibited better catalytic efficacy. It is speculated that the change of the catalytic property of eMIONs was due to the increased reaction probability on the catalytic lattice plane as well as the rising temperature in AMF.

### Magnetic and catalytic study of eMIONs in vitro

**Fig. 4:**
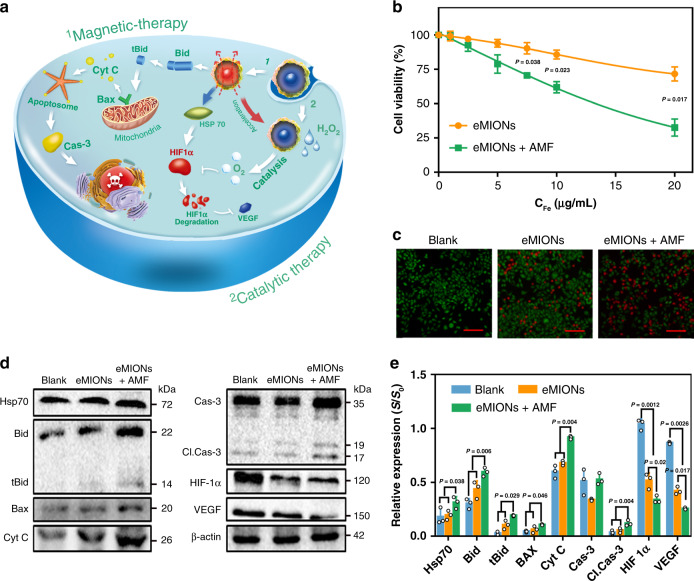
In vitro studies of encapsulin-produced magnetic iron oxide nanocomposites (eMIONs). **a** Schematic illustration of magneto-catalytic therapy by eMIONs and proposed cellular pathways. **b** Viabilities of A549 cells receiving different concentrations of eMIONs with/without alternative magnetic field (AMF). Data are presented as mean ± SD (*n* = 5 biologically independent samples). **c** Fluorescence image of AM/PI stained A549 cells after therapy. Scale bar: 50 μm. **d**, **e** Detection of apoptosis and HIF-1α-related pathways in A549 cells after different treatments by western blot (**d**) and its semiquantitative analysis (**e**). The samples derive from the same experiment and that blots were processed in parallel. Data are presented as mean ± SD (*n* = 3 biologically independent samples). One of three repetitions with similar results is shown for (**c**) and (**d**). Statistical significance was calculated with two-tailed Student’s *t* test (**b**, **e**), with *P* values showed in the figures.

Molecular MRI provides a promising tool to test the cell–eMION interactions^25^. The cell-internalized eMIONs shortened the spin–spin relaxation time and resulted in obviously decreased signal intensities on *T*_2_-weighted images (Supplementary Fig. 7a). Next, cytotoxicity of encABC and eMIONs were studied in a series of cell lines by MTT assay (Supplementary Fig. 7b, c). Interestingly, we found that the cell viabilities of all the normal cells (293T and LO2) were >90%, which were much higher than those of the tumor cells (below 80%, where the A549 cells were 68.7%). The result indicated a significant difference that denotes tumor-specific toxicity caused by the catalysis of eMIONs. We detected the concentration of H_2_O_2_ in these cell lines with or without eMIONs. The tumor cell lines had over two times higher levels of H_2_O_2_ than normal cells (Supplementary Fig. 7d), and eMIONs could catalyze ~50% of H_2_O_2_ in tumor cells. Thus, we proposed that eMIONs could provide cancer therapy through magnetic hyperthermia and catalytic therapy^26–30^ (Fig. 4a). As expected, eMIONs without/with AMF (15 kA/m, 300 kHz, and 10 min) killed almost 35%/65% of the treated A549 tumor cells after 24-h incubation (Fig. 4b, c), and a similar finding was observed in LM3 tumor cells (Supplementary Fig. 8), whereas the fMIONs showed no obvious effect (Supplementary Fig. 9). Afterward, we investigated the cell-damage mechanism caused by MCT. The results revealed that HSP 70, Bid, and cleaved caspase-3 were activated by MHT, reflecting the onset of apoptosis (Fig. 4d, e). Moreover, the expression of HIF-1α and VEGF was sharply declined by 52.8 and 51.1% after being incubated with eMIONs, and further reduced by 32.8 and 45.2% with AMF, respectively. These results showed that eMIONs could generate heat, induce apoptosis, and synergize its catalyzation to realize MCT effects.

### In vivo MCT based on eMIONs

**Fig. 5:**
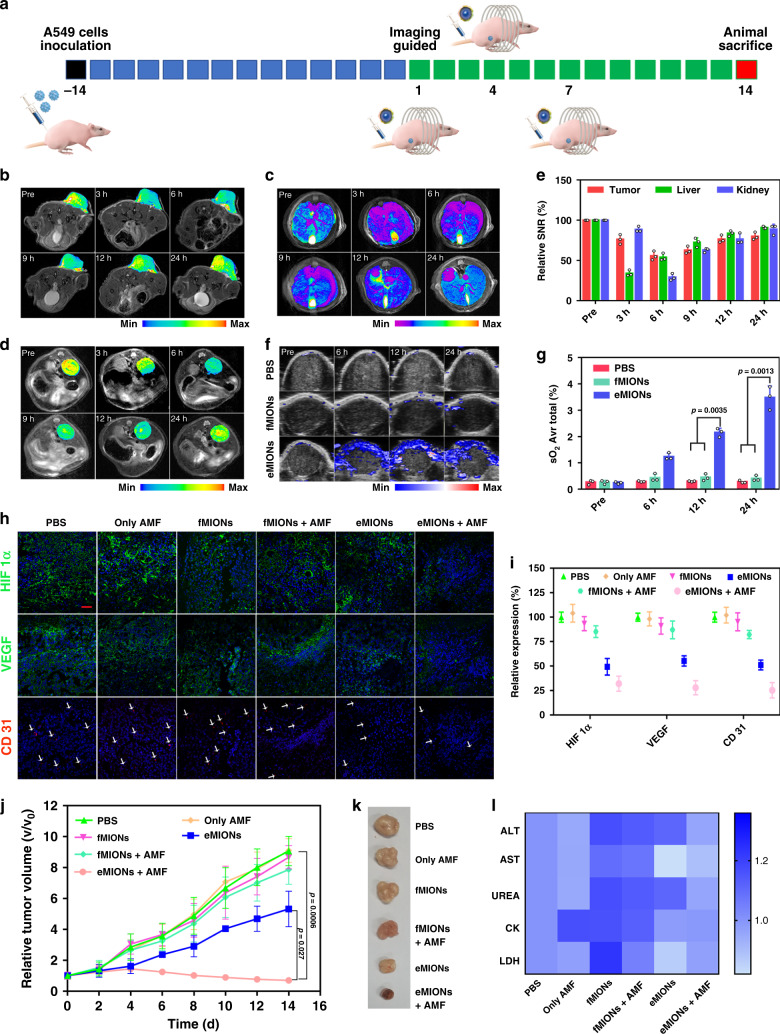
Subcutaneous tumor model theranostics by encapsulin-produced magnetic iron oxide nanocomposites (eMIONs). **a** Schematic illustration of animal therapy by eMIONs. **b**–**e** magnetic resonance imaging (MRI) images with heatmap of the tumor (**b**), liver (**c**), and kidney (**d**) at different time points after injection and semiquantitative analysis (**e**). Data are presented as mean ± SD (*n* = 3 biologically independent mice). **f**, **g** PA-US imaging detected oxyhemoglobin saturation of tumor region after injection (**f**) and semiquantitative analysis (**g**). Data are presented as mean ± SD (*n* = 3 biologically independent mice). **h**, **i** Expression of HIF-1α, VEGF, and CD 31 in tumor regions were detected by immunofluorescent staining after different treatments. One of the three repetitions with similar results is shown here. Scale bar: 20 μm. **j**, **k** Tumor growth curves (**j**) and representative tumors (**k**) showing significantly improved antitumor efficacy of eMIONs with AMF compared with other groups. Data are presented as mean ± SD (*n* = 5 biologically independent mice). **l** Liver and kidney function of mice were detected after treated by different means. Statistical significance was calculated with two-tailed Student’s *t* test (**g**, **j**), with *P* values indicated in the figures.

In order to minimize the adverse effects and maximize the therapeutic effect, MRI and ICP-MS assay were applied to monitor the in vivo behavior of eMIONs (C_Fe_ 2 mg/kg) in an A549 subcutaneous xenograft model after i.v. injection (Fig. 5a–d). Both MRI and ICP-MS results showed that eMIONs were eliminated effectively from the liver and kidneys, but maintained at high levels in the tumor because of the tumor EPR effect and 9 h post injection appears to be the optimal time point for therapy intervention when the tumor accumulation reaches the peak value (Fig. 5e and Supplementary Fig. 10). To monitor the oxyhemoglobin saturation changes in the tumor region, PA-US imaging was used to evaluate the catalytic efficiency after i.v. injection of eMIONs (Fig. 5f). It was found that the tumor O_2_ level of eMIONs group was about 13 times higher than that of the PBS group (Fig. 5g).

The MCT antitumor effects were further evaluated in A549 tumor-bearing mice. As shown in Fig. 5a, the AMF (15 kA/m, 300 kHz, and 10 min) was performed at 9 h post injection, and tumor growth rates were monitored every 2 days to assess the therapeutic effects. A randomly chosen mouse from each group was sacrificed to harvest tumor tissues for further immunofluorescence (IF) examination. The IF results demonstrated that the expression of HIF-1α, VEGF, and CD 31 was significantly downregulated in eMIONs group and was much lower in eMIONs + AMF group compared with the other groups, showing that the catalytic effect changed the tumor microenvironment to inhibit angiogenesis (Fig. 5h, i). As expected, the A549 tumor growth in eMIONs + AMF group was greatly inhibited, and the tumor volume was even reduced 93% compared to that of the PBS group. In addition, eMIONs group offered much better inhibition (46%) than fMIONs because of their efficient catalysis to inhibit tumor angiogenesis (Fig. 5j, k). The H&E staining results of tumors also showed a similar trend of therapeutic effect (Supplementary Fig. 11a). The systemic toxicity of eMIONs was measured via hematological parameters, body weight, and H&E analysis of major organs (Supplementary Fig. 11b–d). The biochemical indexes of the liver and kidney function, such as alanine transaminase (ALT), aspartate aminotransferase (AST), lactic dehydrogenase (LDH), creatine kinase (CK), and urease (UREA) of all groups were within normal ranges (Fig. 5l). Furthermore, no obvious body weight changes and organ damage were observed in any of the treated groups.

**Fig. 6:**
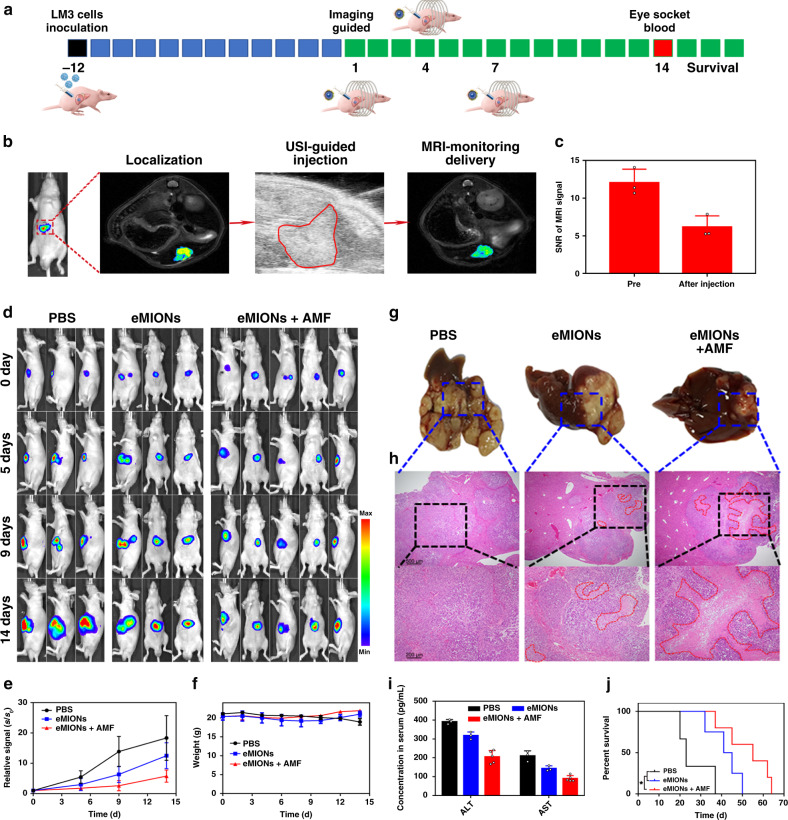
Orthotopic hepatocellular carcinoma (HCC) model theranostics by encapsulin-produced magnetic iron oxide nanocomposites (eMIONs). **a** Schematic illustration of animal therapy by eMIONs. **b** Orthotopic HCC model verified and multimodality imaging-guided eMION delivery. **c** Semiquantitative analysis of magnetic resonance imaging (MRI) before/after eMIONs injection. Data are presented as mean ± SD (*n* = 3 biologically independent mice). **d** Bioluminescence imaging of LM3 tumors in response to eMIONs treatment with/without alternative magnetic field (AMF). **e**, **f** Growth curves for tumors (**e**) and body weight changes of mice (**f**) with different treatments. Data are presented as mean ± SD (Group PBS and eMIONs, *n* = 3 biologically independent mice, and group eMIONs + AMF, *n* = 5 biologically independent mice). **g**, **h** Representative orthotopic HCC (**g**) and H&E stain results at 2 weeks after therapy (**h**). Scale bar of the above pictures: 500 μm; scale bar of neither pictures: 200 μm. **i** Liver function was detected 2 weeks after therapy. **j** Survival curves of mice after therapy (*n* = 5 biologically independent mice). Statistical significance was calculated with two-tailed Student’s *t* test (*P* = 0.047), and * indicated that *P* < 0.05.

To further assess the capacity of eMIONs for deep-seated tumor therapy, we carried out MCT on BALB/c nude mice bearing fLuc-LM3 orthotopic HCC (Fig. 6a). As shown in Fig. 6b, the orthotopic HCC model was detected and monitored by bioluminescence imaging, MRI, and ultrasound imaging (US). The changed *T*_2_ signals revealed the homogenous distribution of eMIONs in HCC with US-mediated intratumoral injection (Fig. 6c). After three times of treatments, quantitative bioluminescence measurements of orthotopic HCC among the different groups demonstrated a significant difference in tumor area (Fig. 6d, e). Tumor growth rates in the eMIONs + AMF and eMIONs groups were decreased by 68.5 and 31.8% compared to the PBS control group, respectively. These results revealed that AMF played a key role in locoregional HCC treatment due to its hyperthermia and promotion for catalysis therapy. During 14 days of therapy, the body weight of the mice showed no obvious changes among all of the groups (Fig. 6f), indicating no severe adverse effects from this therapy.

Next, a randomly chosen mouse from each group was sacrificed to harvest tumor tissue and main organs for further H&E staining to visually observe the efficiency of MCT at day 14. As shown in Fig. 6g, the tumor from the eMIONs + AMF group was much smaller than the other groups. The H&E staining (Fig. 6h and Supplementary Fig. 12) showed that eMIONs suppressed tumor invasion to a certain degree compared with the control group, and tumor cells were killed more in the eMIONs + AMF group, which inhibited its invasion but with no obvious systemic toxicity. Blood from the eye socket was obtained to measure liver function. As shown in Fig. 6i, liver function of eMIONs + AMF group was slightly impacted compared to that in the PBS group, and demonstrated that eMIONs are effective for HCC therapy. Furthermore, the survival curve of these mice showed that the mice survival time of eMIONs + AMF group nearly double compared to the PBS group (Fig. 6j), demonstrating that eMIONs could be excellent MHT agents to suppress orthotopic tumor growth under guided imaging due to its catalysis and magnetic properties. Taken together, eMIONs could overcome the obstacle of MHT with MRI monitoring to guide the treatment and show safe and high therapeutic efficacy.

## Discussion

Benefitting from the capacity of minimally invasive and local hyperthermia ablation of deep-seated lesions, MHT using IONs as heat mediators has been used in the clinic, such as anticancer therapy^31^. It is strongly desirable to develop high-performance IONs to improve MHT efficacy and the safety profile of IONs. In this work, we have addressed the fundamental principles of eMIONs as MHT agents and basics of magnetic hyperthermia, including the nanoparticle size, composition, magneto-crystalline anisotropy, and magnetization effects, on the magnetic-heat-induction ability of eMIONs. EncapsulinABC magnetic protein nanocages were genetically engineered to produce eMIONs with superior magnetic responsiveness via a biosynthetic procedure. The degree of crystallinity of eMIONs reaches up to ~100%, possibly due to the high expression quantity of inner-function proteins encB and encC. Interestingly, we demonstrate that, in the structure of eMIONs, Fe_3_O_4_ has FeO subdomains, which might form Fe^2+^-deficient Fe_3_O_4_ within AMF because of the instability of FeO^32^. Also, we reveal that eMIONs are multi-domains nanoparticles, which have magnetic domains and remnant magnetization that could produce coercive force via hysteresis losses in the presence of magnetic field, and that leads to significantly improved energy-dissipation rate to enhance the magnetic-to-thermal conversion capacity. Thus, the SAR value of eMIONs is as high as 2390 W/g, which is over 20 times higher than that of Feridex (FDA approved IONs)^5^. Taken together, it is not surprising that eMIONs are able to cause a rapid increase in temperature both in vitro and in vivo under AMF.

Furthermore, the mechanism reveals that eMIONs are catalase-like nanozyme in the mild acidic environment, and the catalytic efficiency could be significantly improved within AMF. Therefore, eMIONs could catalyze endogenous H_2_O_2_ into O_2_ to regulate TME, leading to angiogenesis inhibition with a significant decrease in the expression of HIF-1α^27–29^. As a result, the superior magnetic reactors achieved MCT to inhibit tumor growth and extend the survival time of orthotopic HCC-bearing mice. To the best of our knowledge, this is the first demonstration of MCT antitumor efficacy with eMIONs that is an ideal MCT agent suitable for the treatment of deep solid tumors.

Although many chemical synthetic XIONs (X = Mn, Co, Mg, etc.) show excellent magnetic-to-thermal capacity, random size distribution, and high manufacturing cost limit their clinical translation^11,33^. In this work, inspired by the advantages of high protein yield, simple procedures, and low price in *Escherichia coli* (*E. coli*) expression system^34^, we obtain eMIONs with favorable homogeneity, showing that the unique scaffold of protein nanocages have been used in clinical practice, Hecolin is the first nanocage-based vaccine against hepatitis E virus, which licensed by the State Food and Drug Administration of China^35^. Another example is Aβ1-6 peptide coupled RNA bacteriophage Qβ nanocage, which has been in Phase 2 clinical trials for Alzheimer’s disease^36^. Therefore, the *E. coli* recombinant natural iron-storage magnetic protein nanocages are suitable for a translational pipeline, although more toxicological evaluation is needed.

Overall, eMIONs have achieved tumoral precise diagnosis and imaging-guided effective therapy in subcutaneous and deep-orthotopic models, while continued studies to improve specificity for MCT are being explored. For example, active targeting ligands, including peptides and antibodies, could be introduced to eMIONs via genetic engineering technology for better tumor accumulation. Apart from this, other robust protein engineering strategies, such as metal ion-assisted interface re-engineering technology^37^, can be used to expand the biofunctions of eMIONs, providing an optimal condition as a nanoreactor for catalysis and magnetism properties. Furthermore, eMIONs have the potential to be a favorable magnetic particle imaging (MPI) agent due to the excellent magnetization and optimal size^38,39^, and the efforts are now underway in our laboratory. Notably, eMIONs also can be extended to coordinate with other chemotherapeutic agents that can work in combination with MHT to achieve more comprehensive therapeutic results.

In summary, inspired by the natural iron-storage protein, we have genetically recombined and biomimetically mineralized a magneto-protein nanocage as a unique, powerful MHT agent, which integrates potent magnetic-to-thermal conversion efficiency, magnetic-enhancing catalysis, and excellent MRI ability into a single particle to surmount the clinical challenges of MHT. Such a simple and valuable bioinspired strategy will not only provide new insight into MHT development but also can be extended to therapeutics for other diseases, such as diabetes by precise regulation of glucose metabolism via heat-mediated signal transduction in cells^40,41^.

## Methods

### Materials

BCA protein assay kit (#23225) and Isopropylthio-β-D-thiogalactopyranoside (IPTG, #R0392) were purchased from Thermo Fisher Scientific (USA). β-actin (#ab 8226), HSP 70 (#ab 5439), Bid (#ab 10640), Cyt C (#ab 133504), VEGF (#ab 32152), and Caspase-3 (#ab 13847) antibodies were purchased from Abcam (USA). Bax (#YM 3619) and HIF-1α (#YT 2133) antibodies were purchased from ImmunoWay Biotechnology, Inc (USA). Calcein-AM and propidium iodide assay kit (#04511) and MTT (#M2128) were obtained from Sigma-Aldrich (USA).

### Expression and purification of recombinant encapsulinABC

Gene sequence of encA (MXAN_3556), encB (MXAN_3557), and encC (MXAN_4464) were chosen from GenBank, and constructed into vector pRSFDuet-1 (encA) or pCDFDuet-1 (encB and encC), respectively, by Sangon Biotech (Shanghai), Co., Ltd, China. A single colony of *E. coli* BL 21 (DE 3) cells, transformed with protein expression plasmid, was transferred into 5 mL LB medium, supplemented with 100 ng/μL antibiotic, and incubated overnight at 37 °C with 180 rpm shaking. The overnight preculture was then inoculated into 500 mL LB medium and incubated at 37 °C with 180 rpm shaking. Recombinant protein production was induced at OD_600_ = 0.6 by the addition of 1 mM IPTG for another 4-h incubation. Cells were pelleted by centrifugation at 7000 × *g* for 10 min at 4 °C, and resuspended in tenfold (volume per gram of cell pellet) in lysis buffer for sonication on ice with 2-s burst of sonication at 40% amplitude and 4-s of cooling, the total time of sonication is 7 min. Cell lysate clarified by centrifugation at 10,000 × *g*, 10 min, 4 °C, and then polyethylene glycol added to the supernatant on ice and mixed for 45 min, after which precipitated proteins were pelleted. The pellets were resuspended in PBS (pH 7.0) on ice for 1 h, and then centrifuged at 10,000 × *g*, 10 min, 4 °C to remove the sediment. The supernatant was filtrated using 0.22-μm syringe filter (Millipore, UK) for molecular sieve (AKTA Purifier, GE). The collected proteins were verified by SDS-PAGE, dynamic light scatter (DLS), and cryo-electron microscope (Cryo-EM), and then stored at 4 °C.

### Preparation of eMIONs and fMIONs

First, a radius flask (50 mL) with a stirrer was filled with Argon gas, then added a degassed NaCl solution (pH 8.5, 8.0 mL, 100 mM) and encABC (2 mg) with stirring of 1000 rpm and 50 °C. Next, Fe^2+^ (125 mM, FeSO_4_·6H_2_O) and fresh H_2_O_2_ (41.7 mM) were slowly added into the vessel (5 μL/min) by syringe simultaneously. In total, 50 mM NaOH was titrated into a vessel to keep pH to 8.5. For the fMIONs, ferritin (2 mg) was added into an Argon gas-filled radius flask, followed by the addition of 8 mL degassed 100 mM NaCl solution to the flask. Next, Fe^2+^ (12.5 mM, FeCl_2_·4H_2_O) and fresh H_2_O_2_ (4.17 mM) were injected into the vessel (31.3 μL/min) by syringe simultaneously. In all, 50 mM NaOH was titrated into a vessel to keep pH to 8.5. The reacted temperature was kept at 65 °C, and with 1000 rpm stirring for 1 h. After the reaction, the solution purified via ultrafiltration (30 KDa, 2000 rpm/30 min), and then resuspended in 2 mL PBS.

### Characterization of eMIONs and fMIONs

Then, the collected eMIONs and fMIONs were characterized by DLS (Malvern Instruments, Malvern, UK), TEM (TecnaiG2 Spirit, FEI), BCA kit (Thermo Fisher), Inductively Coupled Plasma Mass Spectrometer (ICP-MS). The crystallinity and composition of samples were measured by X-ray diffraction (XRD, Rigaku Ultima IV, Japan) and X-ray photoelectron spectroscopy (XPS). Magnetic characterizations of the samples were represented by a vibrating-sample magnetometer (VSM) option under a magnetic field up to 20 kOe at 5 K. The *T*_*2*_-weighted MR imaging ability was performed with a 9.4-T MRI scanner (Bruker, USA).

### Specific absorption rate (SAR) measurement

The magnetic-to-thermal conversion capacity of eMIONs was measured by SAR-based time-dependent calorimetric measurements under AMF. eMIONs with a series of concentration (0.1, 0.2, 0.5 mM) were positioned at a circular copper coil (10-turn copper coil with an 8 cm outer diameter). Magnetic field was produced by an AMF generator (SPG-10-II, Shenzhen Double-Power Technology Co. Ltd. China) and with different powers (10, 15, 20 KA/m, 300 kHz). The temperature changes of the samples were measured by a thermocouple thermometer (TES-1310, Taiwan). Finally, the SAR value was calculated using the following Eq. (1)^42^:

1 $${\mathrm{SAR}} = {\mathrm{C}}_{\mathrm{P}}\frac{{{\mathrm{{\Delta}}}T}}{{{\mathrm{{\Delta}}}t}} \times \frac{{m_V}}{{m_{NP}}},$$

where C_p_ is the heat capacity of the medium, Δ*T*/Δ*t* is the experimentally observed heating rate, *m*_*V*_ is the mass of the suspension, *m*_NP_ is the iron content of the suspension.

### Study of catalase-like ability of eMIONs

The oxygen generation and its generated rate in aqueous solutions were used to represent the catalase-like ability of eMIONs. For the catalysis ability detection, the fresh H_2_O_2_ (8.8 M) was diluted to 100 mM by deoxygenated water with different pH (5.5, 6.5, and 7.4), and then 30 mL prepared H_2_O_2_ was injected in a 50 mL of closed three-neck flask with oxygen electrode probe of portable dissolved oxygen meter (Rex, JPBJ-608, China) to measure the concentration of oxygen in solution real time. The eMIONs and fMIONs with different concentrations (0.1, 0.5, 1.0, 2.0, and 5 μg/mL) were injected into the flask by a syringe. Then, the device was placed in a water bath with different temperatures (25, 37, and 43 °C) and AMF to detect the effect of temperature and magnetic field on the catalytic efficiency of eMIONs. Finally, the Michaelis–Menten kinetic curves of eMIONs and fMIONs were gained by plotting the respective initial velocities, and the maximal velocities were calculated by the Lineweaver–Burk plotting with Prism 7.0.

### Cellular experiments

To evaluate the magneto-catalytic therapeutic ability, A549, LM3, U87, 4T1, HUEVC, MSC, LO2, and 293T cell lines were used as cell models and cultured in DMEM (with 10% FBS) at 37 °C with 5% CO_2_. Cells (1 × 10^4^) were seeded on a 96-well plate overnight, then added eMIONs or fMIONs with different concentrations (1.0, 2.5, 5.0, 10.0, 20.0 μg/mL), and closed by aseptic paraffin to culture another 6 h. The medium was replaced with a fresh one and closed by aseptic paraffin, and the hyperthermia groups to receive MHT (15 KA/m, 300 kHz, 10 min), then incubated for another 12 h. After that, a standard MTT assay was utilized to evaluate the cell viabilities (*n* = 5).

For AM/PI staining, calcein-AM and propidium iodide solutions (4 μM) were added into A549 cell lines (3 × 10^5^/well, six-well plate) after different treatments, and then incubated for 30 min, after that, washed three times with PBS carefully for fluorescence image by inverted microscopes (Nikon DS-Ti2).

Western blotting experiments to verify the protein expression of the apoptosis signal path, HSP 70, HIF-1α, and VEGF. A549 cell lines were seeded on a six-well plate (3 × 10^5^/well) to incubate overnight. Then the samples were received different treatments as mentioned above. The cells were harvested for western blotting, and the abundance of proteins was calculated via Image J2x (2.1.4.7, Rawak Software, Inc).

### In vivo MR imaging

Animal experiments were approved by the Animal Care and Use Committee (CC/ACUCC) of Xiamen University. Male BALB/c nude mice (6 weeks, ∼20 g) were obtained from Beijing Vital River Laboratory Animal Technology Co. Ltd. For the subcutaneous xenograft tumor model, 10^6^ A549 cells were injected into the right flank of nude mice, and housing conditions of mice is the specific pathogen-free (SPF) laboratory animal center, the humidity keeps at 40–60% (26–28 °C), and keeps at 10 h of light and 14 h of dark cyclic condition. The mice were used when their tumor volumes reached ~100 mm^3^. The eMIONs (C_Fe_ 2 mg/kg) were injected via tail vein, and MR imaging was executed on a 9.4-T scanner (Bruker, USA) at the scheduled time (0, 3, 6, 12, and 24 h), respectively. *T*_*2*_-weighted MR images were obtained with the following parameters: TR = 2500, TE = 33 ms, field of view (FOV) = 4 × 4 cm, slice thickness = 1 mm, and 19 contiguous slices.

### In vivo magneto-catalytic therapy assisted by eMIONs

For in vivo investigations, when the tumors reach ~100 mm^3^, the mice were randomly divided into six groups (*n* = 5). Then the mice treated with C_Fe_ 5 mg/kg of various formulations at day 1, 4, and 7 through the tail vein. In groups only AMF, fMIONs with AMF, and eMIONs with AMF, the tumors were received with AMF (15 KA/m, 300 kHz, 10 min) at 9 h after injection. For the orthotopic model, we established orthotopic HCC, as described previously. Briefly, a laparotomy executed in the anesthetized nude mouse, and then 25 μL fLuc-LM3 cells (10^7^) were injected into the right liver lobe. After 12 days, bioluminescence imaging by IVIS Lumia II was utilized to screening out tumor-bearing mice, and randomly divided into three groups (*n* = 5). Then the mice treated with C_Fe_ 11.2 μg/per mouse by ultrasound imaging-guided intratumor injection, and monitoring the delivery via MRI at days 1, 4, and 7. After that, in group eMIONs with AMF, the tumors were received with AMF (15 KA/m, 300 kHz) for 10 min. On day 14, we collected about 200 μL blood of the mice from the orbit, and randomly sacrificed a mouse from each group for further H&E staining and immunofluorescence staining.

### Reporting summary

Further information on research design is available in the Nature Research Reporting Summary linked to this article.

## Supplementary information

Supplementary Information

Reporting Summary

## Data Availability

All the other data supporting the findings of this study are available within the article and its supplementary information files and from the corresponding author upon reasonable request. A reporting summary for this article is available as a Supplementary Information file. Source data are provided with this paper.

## Source data

Source Data
