# Supplementary material for: Genetically engineered magnetic nanocages for cancer magneto-catalytic theranostics
Source: Nat Commun. 2020 Oct 27;11:5421. doi: 10.1038/s41467-020-19061-9 (PMC7591490; doi:10.1038/s41467-020-19061-9)
Supplement: Supplementary file 1 — Supplementary Information [file 41467_2020_19061_MOESM1_ESM.pdf]

Supplementary Information

Genetically Engineered Magnetic Nanocages for Cancer Magneto-Catalytic Theranostics

Zhang et *al*.

Table of Contents:

-Supplementary Figures 1-12

Genetically Engineered Magnetic Nanocages for Cancer Magneto-Catalytic Theranostics

Yang Zhang[1], Xiaoyong Wang[1,2], Chengchao Chu[1], Zijian Zhou[3], Biaoqi Chen[4], Xin Pang[1], Gan Lin[1], Huirong Lin[1], Yuxin Guo[5], En Ren[1], Peng Lv[1], Yesi Shi[1], Qingbing Zheng[1,2], Xiaohui Yan[1], Xiaoyuan Chen[3], Gang Liu[1,2*]

[1] State Key Laboratory of Molecular Vaccinology and Molecular Diagnostics, Center for Molecular Imaging and Translational Medicine, School of Public Health, Xiamen University, Xiamen 361102, China.

[2] State Key Laboratory of Cellular Stress Biology, Innovation Center for Cell Biology, School of Life Sciences, Xiamen University, Xiamen 361102, China.

[3] Laboratory of Molecular Imaging and Nanomedicine, National Institute of Biomedical Imaging and Bioengineering (NIBIB), National Institutes of Health (NIH), MD 20892, USA.

[4] Fujian Provincial Key Laboratory of Biochemical Technology, Institute of Biomaterials and Tissue Engineering, Huaqiao University, Xiamen 361021, China.

[5] Fujian Provincial Key Laboratory of Innovative Drug Target Research, School of Pharmaceutical Sciences, Xiamen University, Xiamen 361102, China.

* Corresponding author. E-mail: gangliu.cmitm@xmu.edu.cn

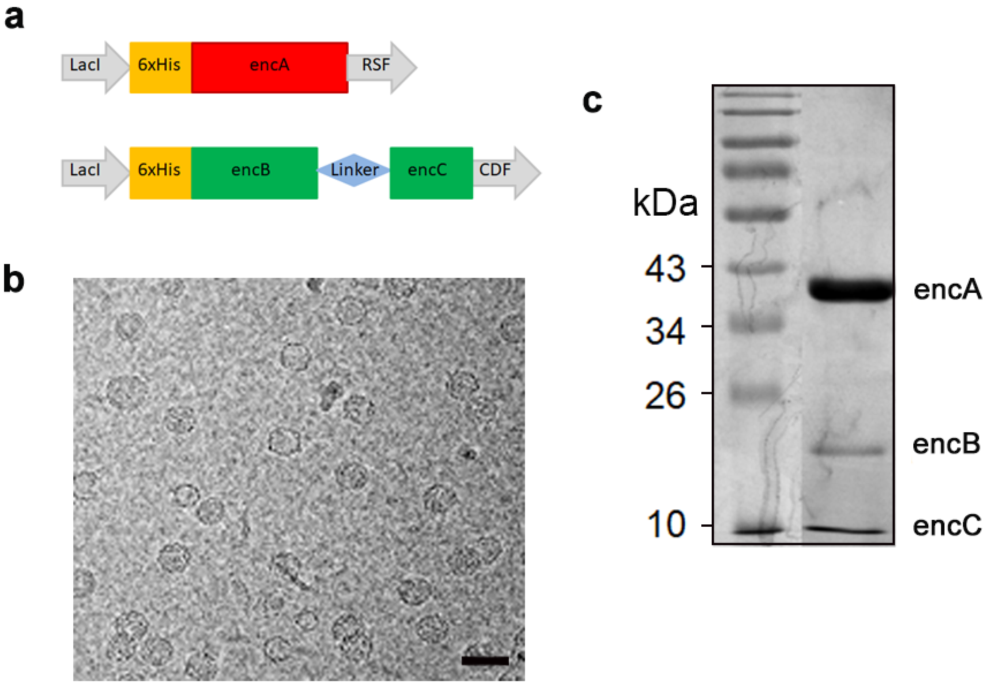

Supplementary Figure 1. Plasmid construction and expression of encABC. **a** encA cloned into pRSFDuet-1; encB and encC cloned into co-expression vector. **b** Representative cryo-EM image of encABC (scale bar: 50 nm). **c** SDS-PAGE to identify the compose of encABC. One of three repetitions with similar results is shown for (**b**) and (**c**).

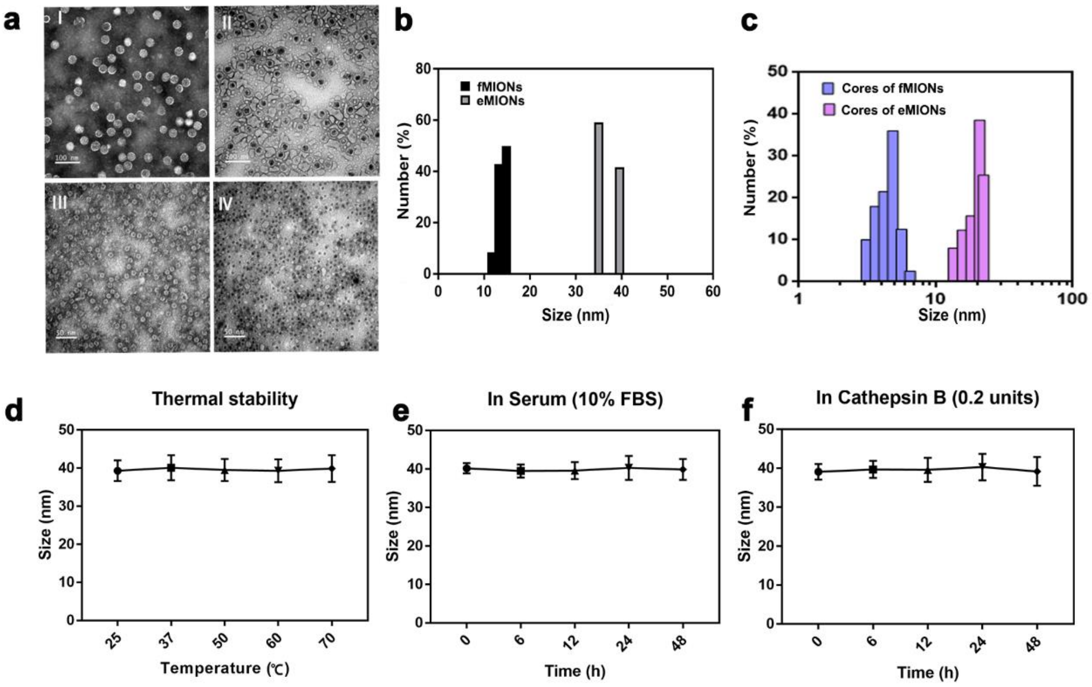

Supplementary Figure 2. Morphology of fMIONs and eMIONs. **a** Representative TEM images of enc ABC (I) (Scale bar: 100 nm), eMIONs (II) (Scale bar: 100 nm) and fn (III) (Scale bar: 50 nm), fMIONs (IV) (One of three repetitions with similar results is shown, Scale bar: 50 nm); **b** Size of the fMIONs and eMIONs. **c** Size of the cores of fMIONs and eMIONs. **d,e,f** Stability detection of eMIONs in different conditions: various temperatures (25 ℃, 37 ℃, 50 ℃, 60 ℃ and 70 ℃) **(d);** in cell culture with 10% FBS at 37 ℃ **(e)**; and in cathepsin B (0.2 units) solution at 37 ℃ **(f).** Data are presented as mean ± SD (n = 5 independent experiments).

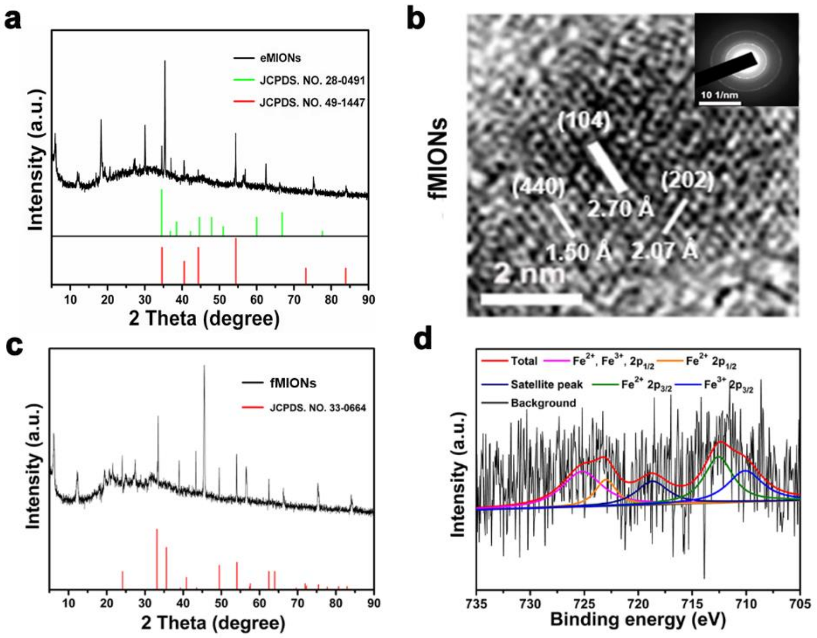

Supplementary Figure 3. Characterization of fMIONs and eMIONs. **a** XRD of eMIONs. **b** HRTEM and corresponding FFT pattern (inset) of fMIONs nanocrystals. One of three repetitions with similar results is shown. **c,d** XRD and XPS analysis to estimate fMIONs, showing its component is γ-Fe_2O_3.

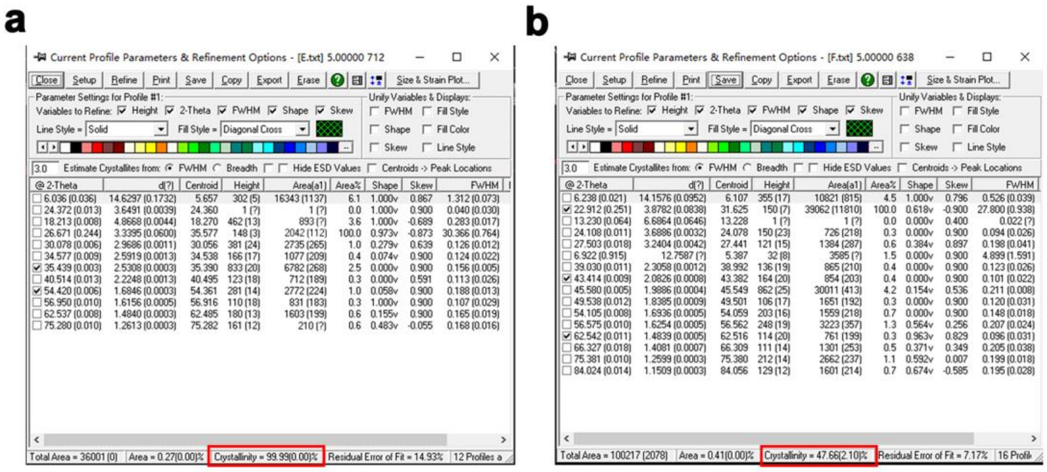

Supplementary Figure 4. Crystallinity evaluation of eMIONs and fMIONs. Results of XRD data analysis by JADE software, showed that the crystallinities of eMIONs (**a**) and fMIONs (**b**) were 99.99% and 47.66%, respectively.

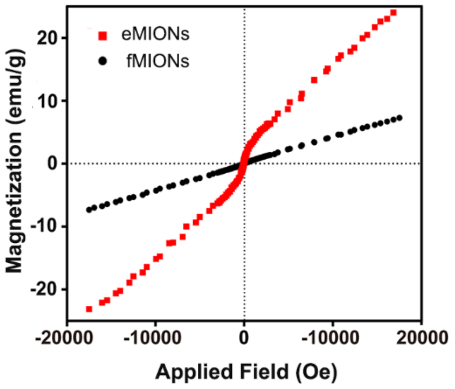

Supplementary Figure 5. Magnetic responsiveness evaluation of fMIONs and eMIONs. Results of VSM analysis of eMIONs and fMIONs.

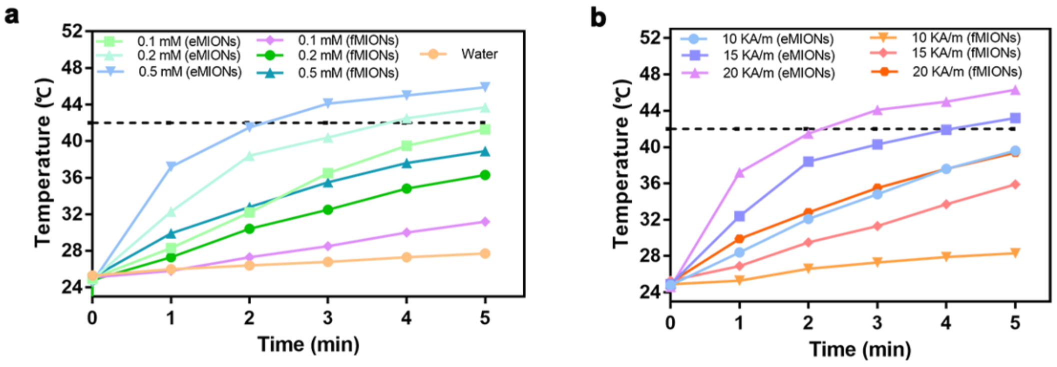

Supplementary Figure 6. Magnetic hyperthermia evaluation of fMIONs and eMIONs. **a** Temperature changes within different concentrations of eMIONs and

fMIONs in AMF (500 kHz, 20 KA/m). **b** Temperature changes within different AMF powers (eMIONs 0.5 mM; fMIONs 0.5 mM). Data are presented as mean ± SD (n = 5 independent experiments).

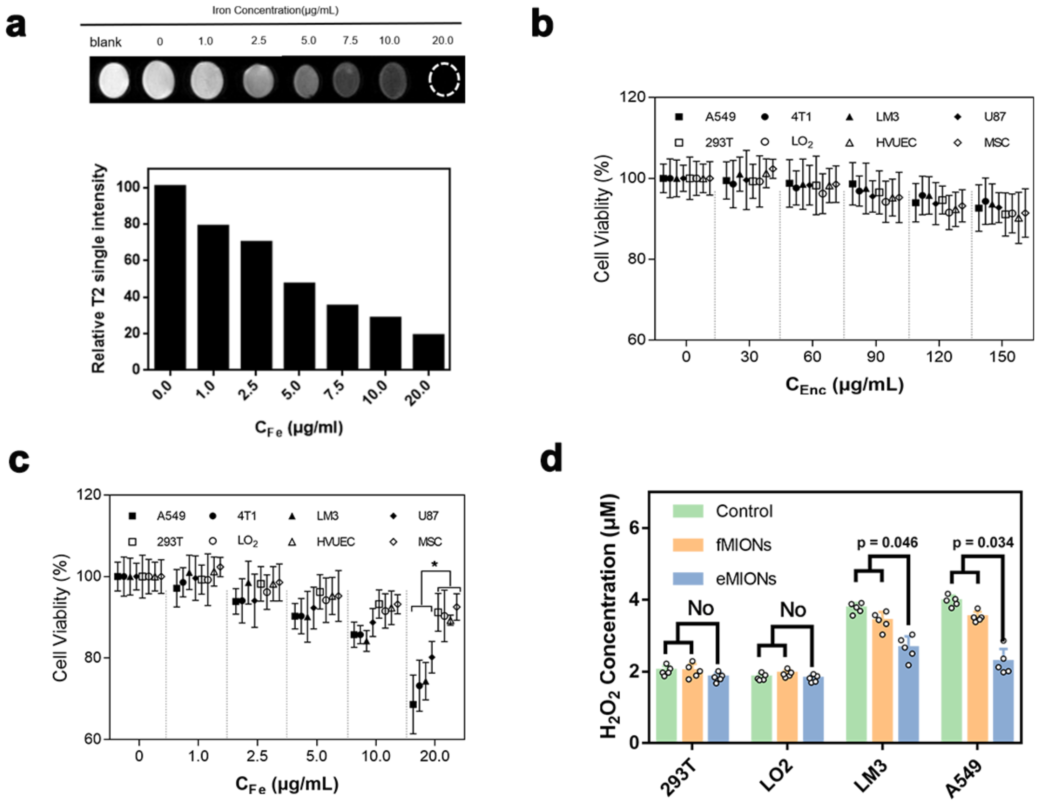

Supplementary Figure 7. Cell uptake and cytotoxicity of NPs. **a** Cell MRI of eMIONs with different concentrations incubated 3 h with A549 cell line. **b** encABC incubated in different cell lines with different concentrations. **c** eMIONs incubated in different cell lines with different concentrations, $p = 0.035$. **d** Concentration of H_2O_2 in different cell lines and after incubated with eMIONs and fMIONs. Data are presented as mean ± SD (n = 5 independent samples), and statistical significance was calculated with two-tailed Student's *t* test, and * indicated that $p < 0.05$.

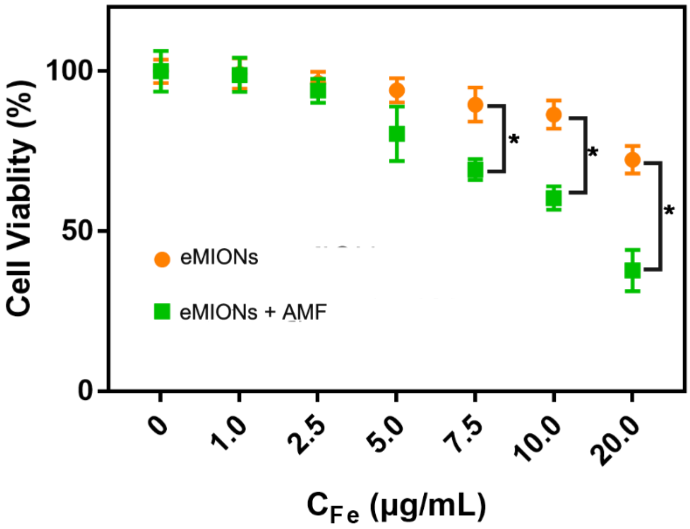

Supplementary Figure 8. Cellular toxicity of LM3 cell line with treatment of eMIONs. Cell viabilities of LM3 cell line receiving different concentration of eMIONs with/without AMF. Data are presented as mean ± SD (n = 5 biologically independent samples). Statistical significance was calculated with two-tailed Student's t test, and * indicated that $p < 0.05$.

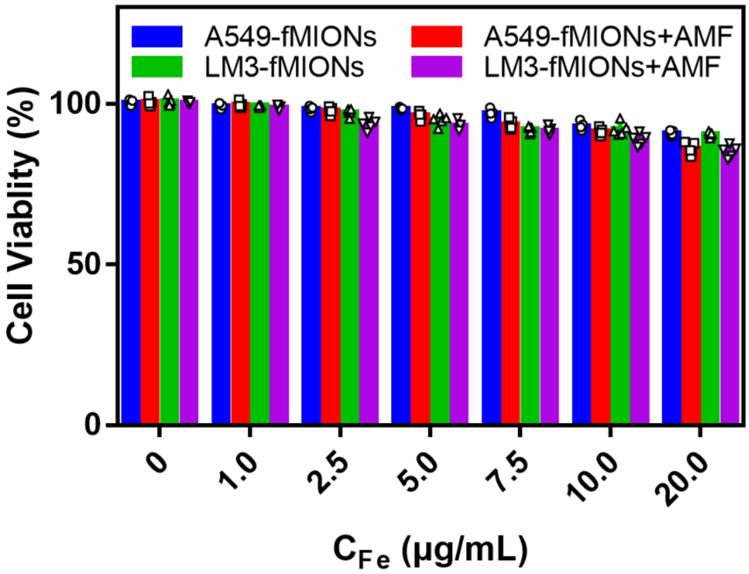

Supplementary Figure 9. Cellular toxicity of A549 and LM3 cell lines with treatment of fMIONs. Cell viabilities of A549 and LM3 cell lines receiving different concentration of fMIONs with/without AMF. Data are presented as mean ± SD (n = 5 biologically independent samples).

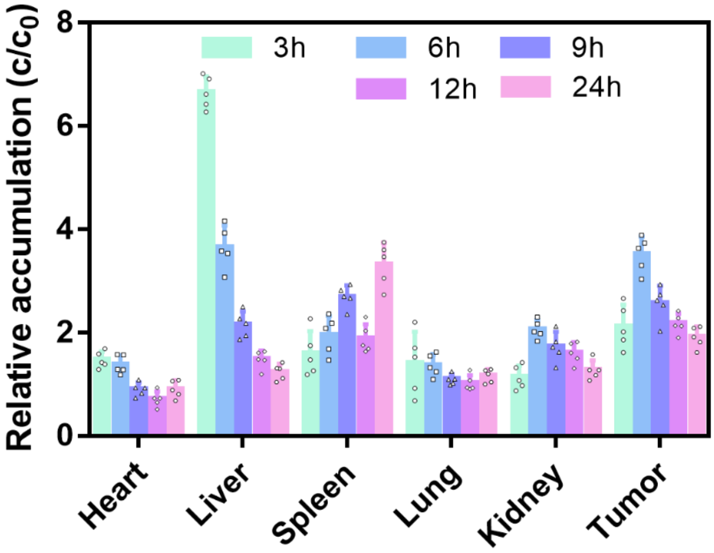

Supplementary Figure 10. Biodistribution of eMIONs. Biodistribution of eMIONs *via* tail vein injection at different time points in main organs and tumor. Data are presented as mean ± SD (n = 5 biologically independent mice).

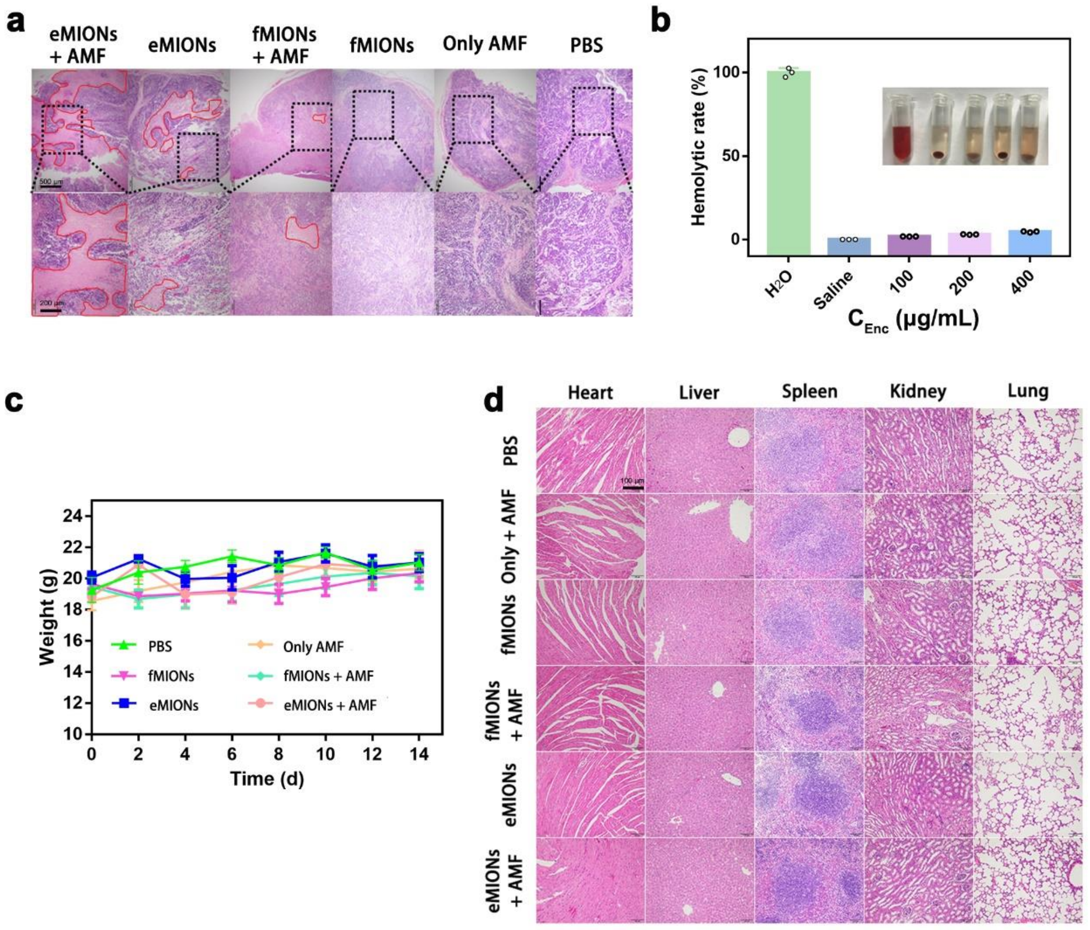

Supplementary Figure 11. H&E staining, hemolytic activity test, and body weight changes of subcutaneous models after therapy. **a** H&E staining of A549 tumor regions at 7 d after different treatments, red solid line shows the regions of undergoing cell death. Scale bar of above pictures: 500 μm; Scale bar of nether pictures: 200 μm. **b** Hemolytic test to verify the biosafety of eMIONs. Data are presented as mean ± SD (n = 3 biologically independent samples). **c** Body weight changing process of mice after therapy. **d** H&E stained major organs harvested from the A549 subcutaneous tumor mice after different therapy, Scale bar: 100 μm. The tumor and all major organs of different groups (n = 5 biologically independent mice) were collected and a slice of each one was stained, the representative images are shown here for (**a**) and (**d**).

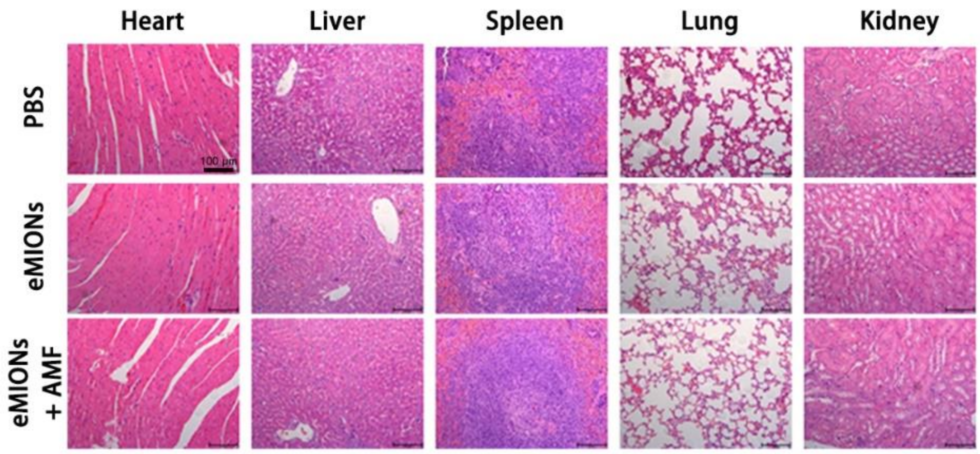

Supplementary Figure 12. H&E staining of orthotopic models after therapy. H&E stained major organs harvested from the orthotopic hepatocellular carcinoma mice after different therapy. Scale bar: 100 μm. The major organs of PBS group (n = 3 biologically independent mice), eMIONs group (n = 3 biologically independent mice) and eMIONs + AMF group (n = 5 biologically independent mice) were collected and a slice of each one was stained, the representative images are shown here.
