# Supplementary material for: Genetically engineered magnetic nanocages for cancer magneto-catalytic theranostics
Source: Nat Commun. 2020 Oct 27;11:5421. doi: 10.1038/s41467-020-19061-9 (PMC7591490; doi:10.1038/s41467-020-19061-9)
Supplement: Supplementary file 2 — Reporting Summary [file 41467_2020_19061_MOESM2_ESM.pdf]

nature research

Corresponding author(s): Gang Liu

Last updated by author(s): Sep 3, 2020

Reporting Summary

Nature Research wishes to improve the reproducibility of the work that we publish. This form provides structure for consistency and transparency in reporting. For further information on Nature Research policies, see our Editorial Policies and the Editorial Policy Checklist.

Statistics

For all statistical analyses, confirm that the following items are present in the figure legend, table legend, main text, or Methods section.

n/a	Confirmed	
☐	☒	The exact sample size (*n*) for each experimental group/condition, given as a discrete number and unit of measurement
☐	☒	A statement on whether measurements were taken from distinct samples or whether the same sample was measured repeatedly
☐	☒	The statistical test(s) used AND whether they are one- or two-sided<br>*Only common tests should be described solely by name; describe more complex techniques in the Methods section.*
☐	☒	A description of all covariates tested
☐	☒	A description of any assumptions or corrections, such as tests of normality and adjustment for multiple comparisons
☐	☒	A full description of the statistical parameters including central tendency (e.g. means) or other basic estimates (e.g. regression coefficient) AND variation (e.g. standard deviation) or associated estimates of uncertainty (e.g. confidence intervals)
☐	☒	For null hypothesis testing, the test statistic (e.g. *F*, *t*, *r*) with confidence intervals, effect sizes, degrees of freedom and *P* value noted<br>*Give P values as exact values whenever suitable.*
☒	☐	For Bayesian analysis, information on the choice of priors and Markov chain Monte Carlo settings
☒	☐	For hierarchical and complex designs, identification of the appropriate level for tests and full reporting of outcomes
☒	☐	Estimates of effect sizes (e.g. Cohen's *d*, Pearson's *r*), indicating how they were calculated

Our web collection on statistics for biologists contains articles on many of the points above.

Software and code

Policy information about availability of computer code

Data collection	TecnaiG2 Spirit, Rigaku Ultima IV, 9.4-T MRI scanner (Bruker)
Data analysis	Statistical analyses were performed on Graphpad Prism 7.0, and Image J2x V2.1.4.7

For manuscripts utilizing custom algorithms or software that are central to the research but not yet described in published literature, software must be made available to editors and reviewers. We strongly encourage code deposition in a community repository (e.g. GitHub). See the Nature Research guidelines for submitting code & software for further information.

Data

Policy information about availability of data

All manuscripts must include a data availability statement. This statement should provide the following information, where applicable:

- Accession codes, unique identifiers, or web links for publicly available datasets
- A list of figures that have associated raw data
- A description of any restrictions on data availability

The source data underlying Figs 3a, e-g; 4b, d, e; 5e, g, j; and 6c, e, i are provided as a Source Data file. All the other data supporting the findings of this study are available within the article and its supplementary information files and from the corresponding author upon reasonable request. A reporting summary for this article is available as a Supplementary Information file.

Field-specific reporting

Please select the one below that is the best fit for your research. If you are not sure, read the appropriate sections before making your selection.

- [x] Life sciences
- [ ] Behavioural & social sciences
- [ ] Ecological, evolutionary & environmental sciences

For a reference copy of the document with all sections, see nature.com/documents/nr-reporting-summary-flat.pdf

Life sciences study design

All studies must disclose on these points even when the disclosure is negative.

Sample size	No effect size was predetermined, but sample sizes employed in this study were consistent with previously published works. For example, in vitro studies were repeated three times independently with triplicate or quintuplicate samples, and in the in vivo experiments with 5 mice per group were performed with three independent replicates. Statistics such as error bars, significance and p values can be derived from n≥3.
Data exclusions	No animals and/or data were excluded.
Replication	All experiments were repeated three times independently and experimental findings were reproducible.
Randomization	Tumor-bearing mice were randomized into the different treatment groups. For the subcutaneous models therapeutics, n = 5 biologically independent mice were divided into each groups randomly. For the orthotopic hepatocellular carcinoma model theranostics, n=3 biologically independent mice in PBS and eMIONs groups, and n=5 biologically independent mice in eMIONs + AMF group. And for the survival detection of orthotopic models, n= 5 biologically independent mice were divided into each group randomly.
Blinding	All the investigators were blinded to group assignment in the course of data collection and analysis.

Reporting for specific materials, systems and methods

We require information from authors about some types of materials, experimental systems and methods used in many studies. Here, indicate whether each material, system or method listed is relevant to your study. If you are not sure if a list item applies to your research, read the appropriate section before selecting a response.

Materials & experimental systems

n/a	Involved in the study	
[ ]	[x]	Antibodies
[ ]	[x]	Eukaryotic cell lines
[x]	[ ]	Palaeontology and archaeology
[ ]	[x]	Animals and other organisms
[x]	[ ]	Human research participants
[x]	[ ]	Clinical data
[x]	[ ]	Dual use research of concern

Methods

n/a	Involved in the study	
[x]	[ ]	ChIP-seq
[x]	[ ]	Flow cytometry
[x]	[ ]	MRI-based neuroimaging

Antibodies

Antibodies used	The following primary antibodies were used for western blotting. β-actin (cat. no. ab8226), HSP70 (cat. no. ab5439), Cyt C (cat. no. ab133504), Bid (cat. no. ab10640), VEGF (cat. no. ab32152) and Caspase 3 (cat. no. ab13847) antibodies were purchased from Abcam (USA); Bax (cat. no. YM3619) and HIF-1α (cat. no. YT2133) antibodies were purchased from ImmunoWay Biotechnology, Inc (USA).
Validation	All antibodies used in this study were validated by the suppliers as follows:<br>β-actin (1:1000, abcam #ab 8226) for WB; Species reactivity (Mouse, Rabbit, Chicken, Cow, Dog, Human, Pig, African green monkey, Chinese hamster, Armenian hamster);Suitable for: ICC/IF, IHC-P, WB;manufacturer's website:https://www.abcam.com/beta-actin-antibody-mabcam-8226-loading-control-ab8226.html<br>HSP 70 (1:1000, abcam #ab 5439) for WB; Species reactivity (Mouse, Rat, Cow, Human, African green monkey); Suitable for: Flow Cyt, ICC/IF, IHC-P, WB, IP; manufacturer's website: https://www.abcam.com/hsp70-antibody-3a3-ab5439.html<br>Bid (1:1000, abcam #ab 10640) for WB; Reacts with: Mouse, Human; Suitable for: WB; manufacturer's website: https://www.abcam.com/bid-cleavage-site-antibody-ab10640.html<br>Cyt C (1:1000, abcam #ab 133504) for WB; Reacts with: Mouse, Rat, Human; Suitable for: WB, IHC-P, ICC/IF, IP; manufacturer's website: https://www.abcam.com/cytochrome-c-antibody-epr1327-ab133504.html<br>VEGF (1:1000, abcam #ab 32152) for WB; Reacts with: Mouse; Suitable for: WB, IHC-P, IP; manufacturer's website: https://www.abcam.com/vegf-receptor-1-antibody-y103-ab32152.html<br>Caspase 3 (1:1000, abcam #ab 13847) for WB; Reacts with: Mouse, Rat, Human, Pig, Xenopus laevis, Drosophila melanogaster, Indian muntjac, Zebrafish, Rhesus monkey; Suitable for: ICC/IF, IHC-Fr, WB, Flow Cyt; manufacturer's website: https://www.abcam.com/caspase-3-antibody-ab13847.html<br>Bax (1:500, ImmunoWay Biotechnology, #YM 3619) for WB; Reactivity：Human,Rat,Mouse,Chicken; Application：IF/ICC,WB,IHC-p;

	manufacturer's website: http://www.bionewway.com/Home/22/YM3619<br>HIF 1α (1:500, ImmunoWay Biotechnology, #YT 2133) for WB; Reactivity：Human,Mouse,Rat; Application：IF/ICC,WB,IHC-p,ELISA; manufacturer's website: http://www.bionewway.com/Home/22/YT2133

Eukaryotic cell lines

Policy information about cell lines

Cell line source(s)	A549, LM3, LO2, U87, 4T1, 293T, HEUVC and MSC cell lines were purchased from Sigma-Aldrich (USA).
Authentication	Cell lines have been authenticated by short tandem repeat profiling, and the results were compared with reference database.
Mycoplasma contamination	All cell lines tested negative for mycoplasma contamination.
Commonly misidentified lines<br>(See ICLAC register)	No commonly misidentified cell lines were used.

Animals and other organisms

Policy information about studies involving animals; ARRIVE guidelines recommended for reporting animal research

Laboratory animals	Balb/c nude mice (male, 6 weeks) were purchased from Beijing Vital River Laboratory Animal Technology Co. Ltd. (Beijing, China).
Wild animals	No wild animals were used.
Field-collected samples	No field-collected samples were used.
Ethics oversight	These studies were performed under a protocol approved by the Animal Care and Use Committee of Xiamen University.

Note that full information on the approval of the study protocol must also be provided in the manuscript.
